# LncRNA GAS8-AS1 suppresses papillary thyroid carcinoma cell growth through the miR-135b-5p/CCND2 axis

**DOI:** 10.1042/BSR20181440

**Published:** 2019-01-11

**Authors:** NingHeng Chen, DeTao Yin, Bing Lun, XueLi Guo

**Affiliations:** 1 General Surgery, The First Affiliated Hospital of Zhengzhou University, Zhengzhou 450052, Henan, China; 2 Obstetrics Department, The Third Affiliated Hospital of Zhengzhou University, Zhengzhou 450052, Henan, China

**Keywords:** GAS8-AS1, CCND2, miR-135b-5p, PTC

## Abstract

The aim of the present study was to investigate the potential role of GAS8 antisense RNA 1 (GAS8-AS1) in papillary thyroid carcinoma (PTC). PcDNA3.1-GAS8-AS1 and si-GAS8-AS1, miR-135b-5p mimic and si-CCND2 were transfected into PTC cells. Cell proliferation was evaluated by Cell Counting Kit-8 (CCK-8). QRT-PCR was used to determine expressions of GAS8-AS1, miR-135b-5p, and CCND2, and Western blot were detected protein level of CCND2. The miRNA target gene prediction site TargetScan was used to predict potential targets of GAS8-AS1 and miR-135b-5p. Cell cycle progression was analyzed by flow cytometry. We found that GAS8-AS1 was down-regulated in PTC cell lines and inhibited proliferation and cycle of PTC cell. GAS8-AS1 directly targets miR-135b-5p, and GAS8-AS1 could regulate a downstream target of miR-135b-5p, Cyclin G2 (CCNG2), in an miR-135b-5p-mediated manner. In addition, we also proved that overexpressed GAS8-AS1 inhibited tumor formation *in vivo*. GAS8-AS1 suppresses PTC cell growth through the miR-135b-5p/CCND2 axis.

## Introduction

Thyroid cancer is the most frequent malignancy of the endocrine system, representing 1–2% of total cancer cases, and the morbidity of thyroid cancer has been substantially increasing over the past few decades all over the world [1,2]. Papillary thyroid cancer (PTC) is the prevalent type of thyroid cancer, and comprises approximately 80% of all thyroid cancers [3]. Regarding the epidemiology of thyroid cancer, more recent data are available that generally support the hypothesis that the increasing incidence of thyroid cancer diagnosis is primarily due to indolent PTC with a relatively stable rise mortality [4]. Most of PTCs could be managed successfully with a combination of adjuvant radioactive iodine therapy and levothyroxine suppression treatment after complete surgical intervention, nevertheless a fraction of patients with PTCs remain irresponsive to treatment and result in comorbidity and mortality. Therefore, further studies on the molecular mechanisms underlying the development and progression of PTC are still urgently needed.

Long non-coding RNAs (lncRNAs) are a group of RNAs, greater than 200 nts in length, which are important for the regulation of gene function and various cellular processes [5]. LncRNAs regulate a variety of processes including cell proliferation, metastasis, invasion migration, apoptosis, development, and differentiation [6]. The growing data have indicated that many lncRNAs are differentially expressed between PTC tissues and adjacent non-cancerous tissues, and are involved in progress of PTC [7–11], but only a small subset have been determined to be biologically significant [12]. GAS8 antisense RNA 1 (GAS8-AS1), which is located in the second intron of GAS8 and transcribes a 994-nt lncRNA in the opposite orientation of GAS8, has been reported to be a novel tumor suppressor that can affect tumor cell proliferation in PTC [13], but the mechanism of how GAS8-AS1 regulates tumor cell function remains unclear.

miRNA, as a class of small non-coding RNA 19–25 nts in length, takes part in post-transcriptional regulation of target genes [14,15]. It has been reported that miRNAs are crucial regulatory factors in numerous cancers [16,17] and numerous cellular processes [18,19]. Mounting evidence have demonstrated that aberrantly expressed miRNAs are involved in regulating development of PTC [20–22]. Previous studies have suggested miR-135b-5p acts as a tumor suppressor, involved in various cancers [23–25]. One study also demonstrated that miR-135b-5p is significantly up-regulated PTC [26]; however, report about the role of miR-135b-5p in the PTC development is in its infancy, and there is no evidence of a relationship between GAS8-AS1 and miR-135b-5p in PTC.

In our study, we indicated that GAS8-AS1 suppresses PTC cell growth through regulation of the miR-135b-5p/CCND2 axis. Therefore, our study has shown that GAS8-AS1/miR-135b-5p/CCND2.

## Materials and methods

### Cell culture and transfection

The human PTC cell line, BCPAP, the German Collection of Micro-organisms and Cell Cultures (Braunschweig, Germany). The human PTC cell line, TPC1 and normal thyroid cell line Nthy-ori-3-1were obtained from Chinese Science Institute (Shanghai, China), and the human PTC cell line, K1, were purchased from the European Collection of Authenticated Cell Cultures (ECACC, U.K.). The human PTC cell line, IHH4, was obtained from Health Science Research Resources Bank (Osaka, Japan). Cell culture medium was prepared according to the suppliers’ instructions. The BCPAP and Nthy-ori-3-1 were cells were cultured in RPMI 1640 medium (Gibco, Carlsbad, CA, U.S.A.) supplemented with 10% FBS (Gibco, Carlsbad, CA, U.S.A.). The TPC1 and K1 were maintained in DMEM high glucose (Gibco, Carlsbad, CA, U.S.A.) supplemented with 10% FBS. IHH4 was maintained in a mixture (1:1) of RPMI 1640 and DMEM high glucose medium supplemented with 10% FBS. All cells were cultured at 37°C, in a humidified atmosphere with 5% CO_2_.

The plasmids of pcDNA3.1 (control) and pcDNA3.1-GAS8-AS1, GAS8-AS1 siRNA (si-GAS8-AS1) and scramble, miR-135b-5p mimic and negative control (NC), CCNG siRNA (si-CCNG), and scramble were obtained from GenePharma (Shanghai, China). TPC1 and BCPAP were transfected using Lipofectamine 2000 (Invitrogen) according to the manufacturer’s protocol.

### QRT-PCR

Total RNA was extracted from the PTC cells and mice tissues using TRIzol reagent (Invitrogen, U.S.A.) following the manufacturer’s protocol. The cDNA was synthesized using a reverse transcription kit. The qRT-PCR was executed with a MiniOpticon real time PCR device. Data were normalized to the reference gene *GAPDH* for each cDNA sample. The primers used in the present study include: GAS8-AS1, 5′-CAACGAGCAAACAAGAAGGAG-3′ (forward) and 5′-TGAGCCAAACAGACCAGTCA-3′ (reverse); miR-135b-5p, 5′-GGTATGGCTTTTCATTCCT-3′ (forward) and 5′-CAGTGCGTGTCGTGGAGT3′ (reverse); Cyclin G2 (CCNG2), 5′-CTTTGGGCATTATTAGGA-3′ (forward) and 5′-GAGGAGGAAACAGTAGCAG-3′ (reverse). The results were calculated by the 2^−ΔΔ*C*^_t_ method.

### Cell proliferation

The proliferation rates of TPC1 and BCPAP were measured using the Cell Counting Kit-8 (CCK-8; Dojindo Laboratories, Kumamoto, Japan). Approximately 2 × 10^3^ cells were seeded in each well of a 96-well plate, and 10 µl of CCK-8 was added to 90 µl of culture medium at the indicated time, and were then transfected with pcDNA3.1-GAS8-AS1, si-GAS8-AS1, miR-135b-5p mimic, and si-CCNG. Proliferation was evaluated at 0, 24, 48, and 72 h after gene transfection. The UV absorbance at 450 nm was measured using a BioTek synergy H1 hybrid reader.

### Cell cycle analysis

TPC-1 and BCPAP cells were transfected with pcDNA3.1-GAS8-AS1, si-GAS8-AS1, miR-135b-5p mimic, and si-CCNG, and analyzed by flow cytometry after 48 h. Briefly, cells were harvested in PBS containing 2 mmol/l EDTA, washed once with PBS, and fixed for 2 h in cold ethanol (70%). Fixed cells were washed once in PBS and treated with 40 mg/ml RNase A in PBS for 30 min. They were then washed once in PBS and stained with 50 mg/ml propidium iodide (Roche). Images of the cell cycle were obtained by the FACS Calibur system (Becton-Dickinson, Franklin Lakes, NJ, U.S.A.).

### Luciferase reporter assay

Cells (5 × 10^4^ cells/well) were cultured in a 24-well plate and co-transfected with wild-type (GAS8-AS1-WT, CCNG2-WT) or mutant (GAS8-AS1-Mut, CCNG2- Mut), miR-135b-5p mimic, and mimic NC using Lipofectamine 2000 (Invitrogen) for 48 h. Firefly activity was normalized to luciferase reporter plasmid (pRL-CMV) *Renilla* activity as control of transfection efficiency. The luciferase activities were measured by the Dual-Luciferase Reporter Assay System (Promega, Madison, WI) according to the manufacturer’s instructions.

### Western blotting

Proteins were extracted with RIPA buffer. The protein concentration was determined using a protein assay kit (Bio-Rad). Approximately 30  µg of protein from each sample was separated on a SDS/polyacrylamide gel (10% gel) and transferred on to PVDFs. Membranes were blocked with 5% skim milk and incubated with anti-CCNG2 antibody (Abcam, Cambridge, U.K.) overnight at 4°C followed by incubation with the corresponding secondary antibodies for 1  h at room temperature. Proteins were detected on membranes, after washing in TBST, using Super ECL Plus Detection Reagent (Thermo Fisher Scientific, Carlsbad, CA, U.S.A.).

### Tumor formation in nude mice

Twenty-four male BALB/c nude mice (8-week-old, 210–250  g) were randomly divided into three groups, eight mice in each group. BCPAP cells (1 × 10^7^ cells in 200 μl serum-free medium) treated with lentiviral vector (LV)-GAS8-AS1 were subcutaneously injected into eight nude mice. All mice were housed in a temperature-controlled room (22–24 °C), with a 12–12 h light/dark cycle. Animals were given free access to chow and water. All procedures in the present study were approved by the Animal Care Committee of The First Affiliated Hospital of Zhengzhou University, and all the methods were carried out in accordance with the approved guidelines. At specific times, the mice were killed, and the weight and volume of the tumors as well as expressions of GAS8-AS1, miR-135b-5p, and CCNG2 were determined.

### Statistics

**Figure 1:**
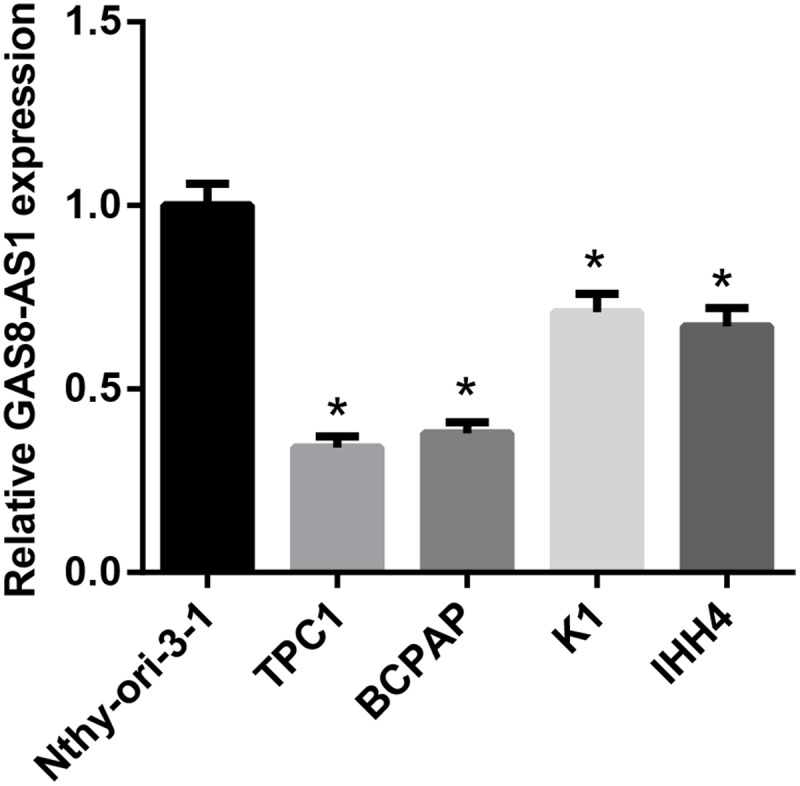
Relative expression of GAS8-AS1 in PTC cell lines and the normal thyroid epithelial cell line Nthy-ori-3-1 was measured by qRT-PCR The level of relative GAS8-AS1 expression was decreased in four PTC cell lines, TPC1, BCPAP, K1, IHH4, compared with Nthy-ori- 3-1 (*P<0.05 compared with Nthy-ori-3-1).

**Figure 2:**
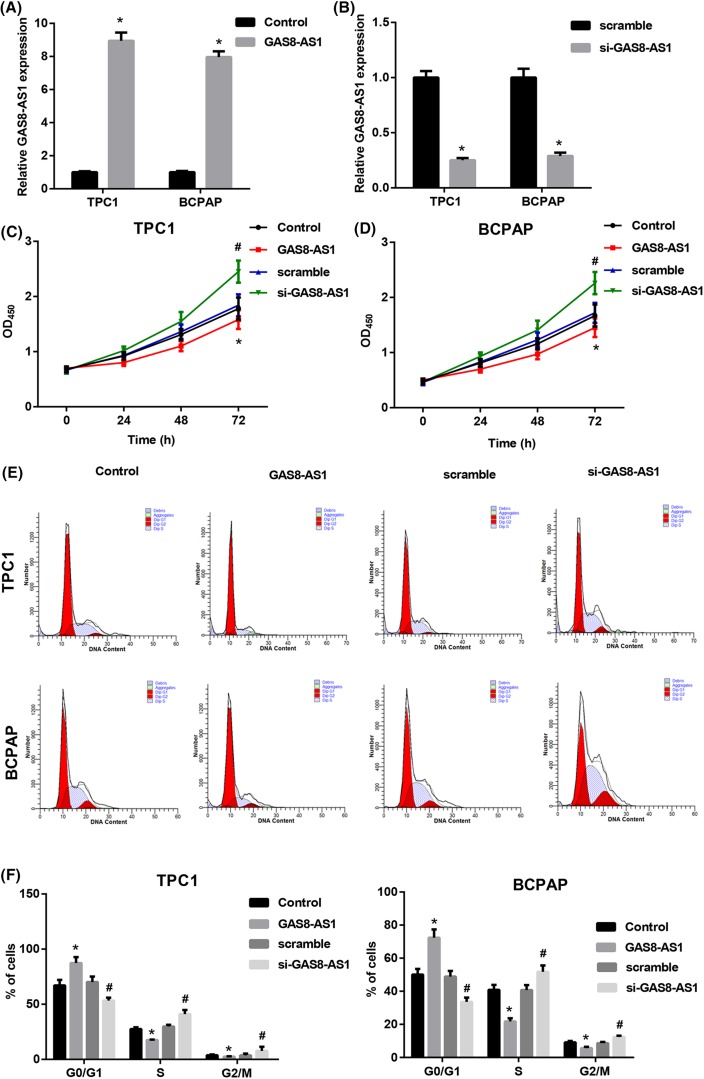
GAS8-AS1 inhibited proliferation in PTC cell growth (**A**) Relative expression of GAS8-AS1 after transfecting pcDNA3.1-GAS8-AS1 (GAS8-AS1 group) and pcDNA3.1 (Control group) into TPC1 and BCPAP was analyzed by qRT-PCR and normalized to GAPDH expression (*P<0.05 compared with Control). (**B**) Relative expression of GAS8-AS1 after transfecting siGAS8-AS1 and scramble into TPC1 and BCPAP was analyzed by qRT-PCR and normalized to GAPDH expression (*P<0.05 compared with scramble). (**C**,**D**) Cell proliferation was performed by CCK-8 assay after transfecting scramble, si-GAS8-AS1, pcDNA3.1, or pcDNA3.1-GAS8-AS1 into TPC1 and BCPAP cells. (**E**) Cell cycle distributions were detected by flow cytometry in treated TPC-1 and BCPAP cells. (**F**) Graphical representation of the cell cycle analysis (*P<0.05 compared with Control; ^#^*P*<0.05 compared with scramble).

**Figure 3:**
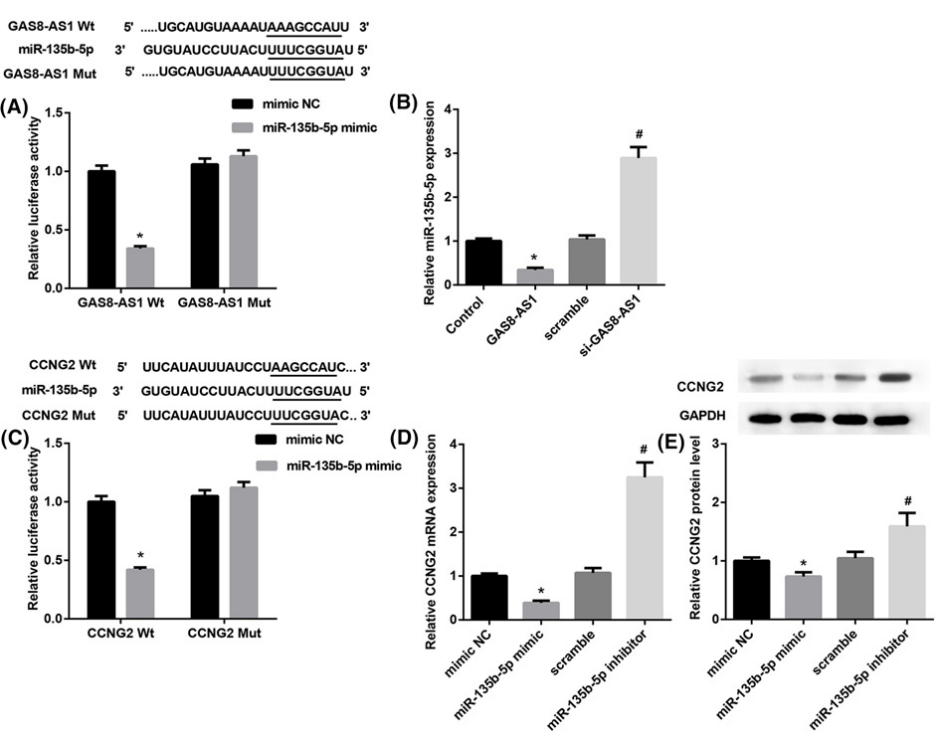
GAS8-AS1 directly targets miR-135b-5p, which targets CCNG2 (**A**) Schematic of the putative GAS8-AS1 target site in human miR-135b-5p 3′-UTR and the eight mutated nucleotides are in lines (*P<0.05 compared with mimic NC). (**B**) QRT-PCR analysis of miR-135b-5p in BCPAP cells transfected with scramble, si-GAS8-AS1, pcDNA3.1, or pcDNA3.1- GAS8-AS1 (*P<0.05 compared with Control; ^#^*P*<0.05 compared with scramble). (**C**) Schematic of the putative miR-135b-5p target site in human CCNG2 3′-UTR and the eight mutated nucleotides are in lines (*P<0.05 compared with mimic NC). (**D**) QRT-PCR analysis of CCNG2 in BCPAP cells transfected with scramble, miR-135b-5p inhibitor, pcDNA3.1, mimic NC, or miR-135b-5p mimic (*P<0.05 compared with mimic NC; ^#^*P*<0.05 compared with scramble). (**E**) Western blotting analysis of CCNG2 in BCPAP cells transfected with scramble, miR-135b-5p inhibitor, pcDNA3.1, mimic NC, or miR-135b-5p mimic (*P<0.05 compared with mimic NC; ^#^*P*<0.05 compared with scramble).

**Figure 4:**
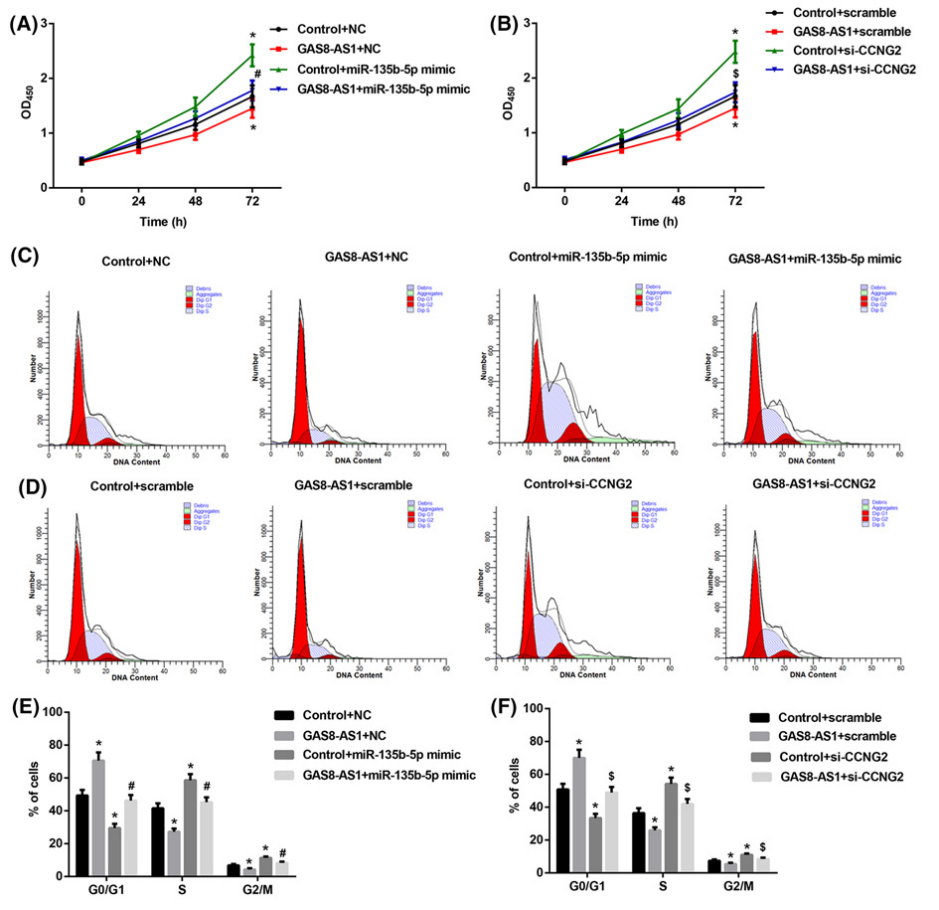
GAS8-AS1 suppresses PTC cell growth through miR-135b-5p/CCNG2 axis (**A**) Cell proliferation was performed by CCK-8 assay after transfecting pcDNA3+miR-135b-5pmimic (Control+miR-135b-5p mimic group), pcDNA3.1-GAS8-AS1+miR-135b-5p mimic (GAS8-AS1+miR-135b-5p mimic group), pcDNA3.1-GAS8-AS1+NC (GAS8-AS1+NC group), or pcDNA3.1+NC (Control +NC group) into BCPAP cells (*P<0.05 compared with Control+NC; ^#^*P*<0.05 compared with Control+miR-135b-5p mimic). (**B**) Cell proliferation was performed by CCK-8 assay after transfecting pcDNA3+si-CCNG2 (Control+si-CCNG2 group), pcDNA3.1-GAS8-AS1+si-CCNG2 (GAS8-AS1+si-CCNG2 group), pcDNA3.1-GAS8-AS1+scramble (GAS8-AS1+scramble), or pcDNA3.1+scramble (Control+scramble) into BCPAP cells. (*P<0.05 compared with Control+scramble; ^$^*P*<0.05 compared with Control+ si-CCNG2). (**C**,**E**) Cell cycle distributions and graphical representation analysis were detected by flow cytometry in treated BCPAP cells with pcDNA3+miR-135b-5pmimic, pcDNA3.1-GAS8-AS1+miR-135b-5p mimic, pcDNA3.1-GAS8-AS1+NC, or pcDNA3.1+NC (*P<0.05 compared with Control+NC; ^#^*P*<0.05 compared with Control+miR-135b-5p mimic). (**D**,**F**) Cell cycle distributions and graphical representation analysis were detected by flow cytometry in treated BCPAP cells with pcDNA3+si-CCNG2, pcDNA3.1-GAS8-AS1+si-CCNG2, pcDNA3.1-GAS8-AS1+scramble, or pcDNA3.1+scramble (*P<0.05 compared with Control+scramble; ^$^*P*<0.05 compared with Control+si-CCNG2).

**Figure 5:**
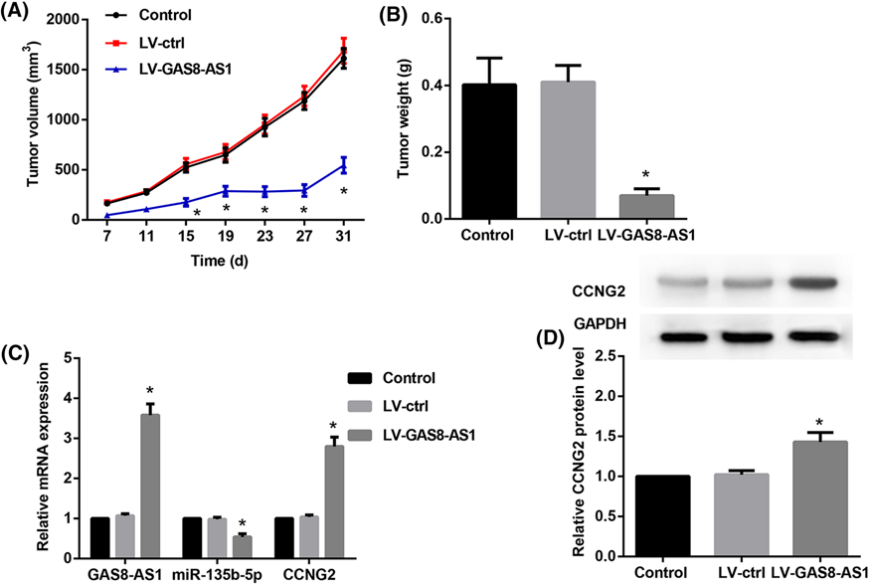
GAS8-AS1 inhibits PTC cell growth through miR-135b-5p/CCNG2 axis in vivo (**A**) Tumors were collected from nude mice injected with BCPAP cells transfected with LV of pcDNA3.1-GAS8-AS1 or control. The tumor volume was analyzed at specific times. (**B**) The tumor weight was measured at specific times. (**C**) Relative mRNA expression of GAS8-AS1, miR-135b-5p, and CCNG2 in tissues from the mice. (**D**) Relative protein expression of CCNG2 in tissues from the mice (*P<0.05 compared with Control or LV-ctrl; *n*=8).

The data are presented as mean ± S.D. Differences between means were determined by Student’s *t*test with in two groups. Differences between more than two groups were analyzed using one-way ANOVA followed by Dunnett’s test (Figures 1–3 and 5) and least significant difference (LSD) test (Figure 4). *P*<0.05 was considered statistically significant. The experiments were repeated three times.

## Results

### Relative GAS8-AS1 expression was reduced in PTC cell lines

We initially examined the relative expression of GAS8-AS1 in four PTC cell lines, TPC1, BCPAP, K1, IHH4, and one normal thyroid epithelial cell line, Nthy-ori-3-1 by qRT-PCR. The mRNA expression of GAS8-AS1 was significantly decreased in all PTC cell lines compared with Nthy-ori-3-1 (Figure 1). The TPC1 and BPCAP (Supplementary Figure S1) were selected for further evaluation because GAS8-AS1 expression in which was decreased by more than 2.0-folds.

### GAS8-AS1 inhibited proliferation in PTC cell growth

PTC cell lines were transfected with GAS8-AS1 overexpression vector (pcDNA3.1-GAS8-AS1) or with siRNA GAS8-AS1 (si-GAS8-AS1), then relative expression of GAS8-AS1 was analyzed by qRT-PCR. The relative expression of GAS8-AS1 in the GAS8-AS1 group (pcDNA3.1-GAS8-AS1) was nine- and eight-folds higher than in the control vector (pcDNA3.1) group in TPC1 and BCPAP cells, respectively (Figure 2A). Relative GAS8-AS1 expression in the si-GAS8-AS1 group was four-folds less than in the non-silencing control (scramble) group in TPC1 and BCPAP cells (Figure 2B). Cell proliferation was detected by CCK-8 assay, si-GAS8-AS1 significantly promoted proliferation compared with the scramble group, and GAS8-AS1 overexpression prominently repressed proliferation compared with the control group in TPC1 (Figure 2C) and BCPAP cells (Figure 2D). To demonstrate the growth-inhibiting effect of GAS8-AS1 on PTC cells, cell cycle progression was analyzed by flow cytometry. As shown in Figure 2E,F, GAS8-AS1 overexpression induced significant increase in the percentage of TPC1 and BCPAP cells at pre-G_1_ relative to control and significant decrease in the percentage of TPC1 and BCPAP cells at S and G_2_/M phases as compared with control. On the contrary, si-GAS8-AS1 induced marked increase in the percentage of TPC1 and BCPAP at S and G_2_/M phases. Conclusively, GAS8-AS1 inhibited PTC cell proliferation and resulted in an interference with the normal cell cycle distribution of PTC cells.

### GAS8-AS1 directly targets miR-135b-5p and miR-135b-5p targets CCNG2

To reveal the molecular mechanism that GAS8-AS1 regulates PTC cell growth, miRNA target gene prediction site TargetScan was used to predict potential targets of GAS8-AS1. Amongst the candidates, we found a highly conservative and specific combination sequence between GAS8-AS1 and miR-135b-5p (Figure 3A). Our results showed that miR-135b-5p mimic significantly repressed luciferase activity when cotransfected with reporter containing WT GAS8-AS1 3′-UTR but not MT GAS8-AS1 3′-UTR (Figure 3A). The synthetic si-GAS8-AS1 or pcDNA3.1-GAS8-AS1 was transfected into BCPAP cells. The result showed that GAS8-AS1 overexpression significantly suppressed mRNA expression of miR-135b-5p, while si-GAS8-AS1 promoted the expression of miR-135b-5p (Figure 3B). Interestingly, we further found evidence that miR-135b-5p might directly target CCNG2. The wild-type and mutant 3′-UTRs of CCNG2 were cloned and inserted into a luciferase reporter vector. We exogenously overexpressed miR-135b-5p via the transfection of cells with a miR-135b-5p mimic. The luciferase activity of the wild-type 3′UTR of CCNG2 was down-regulated in the presence of miR-135b-5p, while the expression of miR-135b-5p did not repress luciferase activity driven by the mutant 3′-UTR of the CCNG2-binding seed region (Figure 3C). Moreover, the mimic or inhibitor of miR-135b-5p was transfected into BCPAP cells. QRT-PCR analysis exhibited that miR-135b-5p mimic significantly suppressed CCNG2 expression (fold change > 3) whereas miR-135b-5p inhibitor repressed CCNG2 expression in mRNA level (fold change > 3) (Figure 3D), CCNG2 protein expression was remarkably suppressed by the miR-135b-5p mimic but enhanced by the miR-135b-5p inhibitor when compared with the NC (Figure 3E). The above results indicated that GAS8-AS1 directly targets miR-135b-5p, which targets CCNG2.

### GAS8-AS1 suppresses PTC cell growth through miR-135b-5p/CCNG2 axis

In our study, we demonstrated the relationships amongst GAS8-AS1, miR-135b-5p, and CCNG2 and their effects on the proliferation and cycle of PTC cell. First, BCPAP cells were transfected with miR-135b-5p mimic and/or GAS8-AS1, si-CCNG2 and/or GAS8-AS1. CCK-8 assay results indicated that GAS8-AS1 repressed the cell proliferation, and this proliferation could be reversed by miR-135b-5p mimic and si-CCNG2 (*P*<0.05) (Figure 4A,B). Furthermore, cell cycle arrest induced by GAS8-AS1, miR-135b-5p mimic and si-CCNG2 in BCPAP cells was evaluated detected by flow cytometry. As shown in Figure 4C–F, compared with control, miR-135b-5p mimic and si-CCNG2 induced significant reduction in the percentage of BCPAP cells at G_0_/G_1_ compared with control and significant increase in the percentage and BCPAP cells at S and G_2_/M phases. Interesting, GAS8-AS1+miR-135b-5p mimic induced significant increase in the percentage of BCPAP cells at G_0_/G_1_ and significant reduction at S and G_2_/M phases compared with control+miR-135b-5p mimic group, while GAS8-AS1+si-CCNG induced similar variation pattern in the comparision with control+si-CCNG group. Therefore, GAS8-AS1 suppresses PTC cell growth through miR-135b-5p/CCNG2 axis.

### GAS8-AS1inhibits PTC cell growth through miR-135b-5p/CCNG2 axis *in vivo*

We further explored the effect of GAS8-AS1 on PTC cell growth *in vivo*. BCPAP cells stably transfected with LV TU/ml of pcDNA3.1-GAS8-AS1 or control were subcutaneously injected into nude mice, and the tumor was excised at 28 days. Tumor volume was smaller in the LV-GAS8-AS1 group than in the control group or LV-ctrl group (*P*<0.05) (Figure 5A). The tumor weight followed the same pattern and was smaller in the LV-GAS8-AS1 group than in the control group and LV-ctrl group (*P*<0.05) (Figure 5B). Our results showed that the expressions of GAS8-AS1 and CCNG2 were markedly increased by 3.5-folds in tissues from mice transfected with LV-GAS8-AS1 compared with control and LV-ctrl group whereas miR-135b-5p expression was 3.5-folds higher than control or LV-ctrl group (Figure 5C,D). To conclude, these observations indicate that GAS8-AS1 repressed the development and progression of PTC through inhibition of miR-135b-5p and activation of CCNG2.

## Discussion

LncRNAs play crucial roles in the development of various types of cancers [27–29], wherein they modulate key cellular processes such as tumor cell proliferation, invasion, and metastasis [30]. GAS8-AS1 is a recently discovered tumor suppressor gene in PTC. GAS8-AS1 expression in plasma was down-regulated in patients with PTC in comparison with those in nodular goiters [31]. A significant depletion of endogenous lncRNA GAS8-AS1 expression was observed in PTC tissue specimens compared with normal tissues of patients [13]. In our study, the expression of GAS8-AS1 was reduced in PTC cell lines compared with a normal thyroid cell line. Overexpressed GAS8-AS1 markedly inhibited proliferation while underexpressed GAS8-AS1 promoted the cell proliferation, and interfered with cell cycle of PTC cells. These findings will provide signifcant contributions to the treatment of PTC. Recently, miR-135b-5p, as an oncogene or tumor suppressor, has been repeatedly reported in cancer-related studies. In these researches, abnormal expression of miR-135b-5p involves in promotion or repression of tumorigenesis and tumor progression by modulating tumor cells proliferation, apoptosis, migration, and so on [25,32]. To determine the mechanism of cell growth induced by GAS8-AS1, we examined the relationship between GAS8-AS1 and miR-135b-5p using miRNA target gene prediction site TargetScan, and found GAS8-AS1 targets miR-135b-5p. In a rescue experiment, we identified the critical role of miR-135b-5p in GAS8-AS1 induced cell growth. Recently, competing endogenous RNA (ceRNA) has been regarded as a new mechanism of post-transcriptional regulation through miRNA competition [33]. CeRNA targets and miRNA can combine to form a complex ceRNA cross-talk, which has pivotal impact on the physiology and development of diseases such as cancer [34]. In this study, the GAS8-AS1 negatively regulated miR-135b-5p in regulating PTC cell growth. However, the ceRNA network is so complex that the GAS8-AS1/miR-135b-5p regulation network in PTC needs intensively study.

CCNG2 is a novel cyclin negatively regulating the cell cycle progression. Several studies indicate that CCNG2 may have an inhibitory role in the progression of cancer as lower expression of CCNG2 is often found in more aggressive cancers [35,36], including thyroid [37]. Therefore, *CCNG2* is often proposed to be a tumor suppressor gene through its regulation of cell proliferation. On the other hand, we found that miR-135b-5p directly targets CCNG2 expression by binding to its 3′-UTR. Our results showed that GAS8-AS1/miR-135b-5p influenced cell proliferation and cycle by regulating the expression of CCNG2, and revealed that CCNG2 could be another therapeutic target in PTC.

In summary, the si-GAS8-AS1 promotes cell growth through overexpression of miR-135b-5p and inhibition of CCNG2. Therefore, our study implies the GAS8-AS1/miR-135b-5p-CCNG2 axis may provide a potential target for PTC treatment.

## Supporting information

**Supplementary Figure S1:** 

## Abbreviations

CCK-8: cell counting kit-8
CCNG2: cyclin G2
ceRNA: competing endogenous RNA
GAS8-AS1: GAS8 antisense RNA 1
lncRNA: long non-coding RNA
LV: lentiviral vector
NC: negative control
PTC: papillary thyroid carcinoma
TBST: TBS containing Tween 20
