# Supplementary figures and images for: LncRNA GAS8-AS1 suppresses papillary thyroid carcinoma cell growth through the miR-135b-5p/CCND2 axis
Source: Biosci Rep. 2019 Jan 11;39(1):BSR20181440. doi: 10.1042/BSR20181440 (PMC6328895; doi:10.1042/BSR20181440)

**A****TPC-1**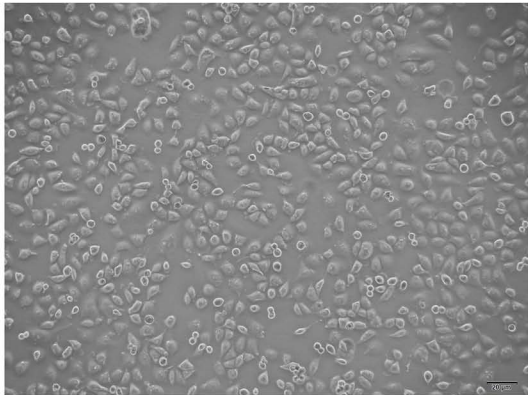**B****B-CPAP**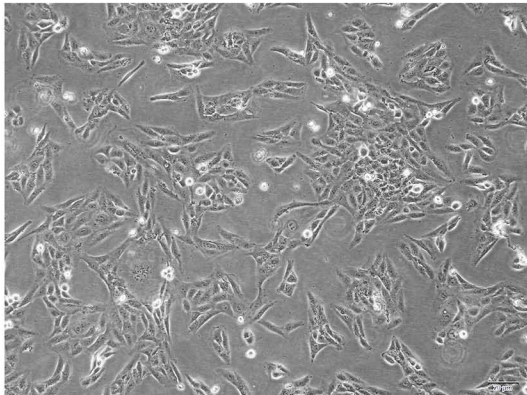

Supplement: Supplementary file 1 [file bsr20181440_Supp1.pdf]
